# Lower CMV and EBV Exposure in Children With Kawasaki Disease Suggests an Under-Challenged Immune System

**DOI:** 10.3389/fped.2020.627957

**Published:** 2021-01-21

**Authors:** Diana van Stijn, Annemarie Slegers, Hans Zaaijer, Taco Kuijpers

**Affiliations:** ^1^Department of Pediatric Immunology, Rheumatology and Infectious Diseases, Emma Children's Hospital, Amsterdam University Medical Center, University of Amsterdam, Amsterdam, Netherlands; ^2^Laboratory of Clinical Virology, Department of Medical Microbiology, Center for Infection and Immunity Amsterdam University Medical Center, University of Amsterdam, Amsterdam, Netherlands

**Keywords:** Kawasaki disease, Epstein-Barr virus, immune system, viral exposure, cytomegalovirus

## Abstract

**Background:** Kawasaki Disease (KD) is a pediatric vasculitis of which the pathogenesis is unclear. The hypothesis is that genetically pre-disposed children develop KD when they encounter a pathogen which remains most often unidentified or pathogen derived factors. Since age is a dominant factor, prior immune status in children could influence their reactivity and hence the acquisition of KD. We hypothesized that systemic immune responses early in life could protect against developing KD. With this study we tested whether the incidence of previous systemic cytomegalovirus (CMV) or Epstein-Barr virus (EBV) infection is lower in children with KD compared to healthy age-matched controls.

**Methods and Results:** We compared 86 KD patients with an age-matched control group regarding CMV and EBV VCA IgG measurements (taken before or 9 months after IVIG treatment). We found that both CMV and EBV had an almost 2-fold lower seroprevalence in the KD population than in the control group.

**Conclusions:** We suggest that an under-challenged immune system causes an altered immune reactivity which may affect the response to a pathological trigger causing KD in susceptible children.

## Introduction

Kawasaki disease (KD) is a rare pediatric vasculitis of unknown etiology that can lead to coronary artery aneurysms (CAAs). These CAAs can result in cardiac complications (1) and even be fatal when an occlusion occurs due to secondary complications such as thrombosis or stenosis (2). If treated properly, i.e., preferably within the first 10 days of illness with intravenous immunoglobulin (IVIG) and oral aspirin for a prolonged period of time, a decrease in the incidence of CAAs has been reported (3, 4). However, with this treatment still ~2.4–27.8% of the KD patients develop CAAs (5–11). To further prevent the development of CAAs due to KD, we need more understanding of the etiology of the disease. Multiple studies suggest that both infectious and genetic factors are involved (12–14). Most likely KD is triggered in genetically pre-disposed children by an intracellular pathogen (15). Several genes have been identified to be associated with KD in children (e.g., ITPKC, CASP3, CD40, and FCGR2A) (16–30). Possible genetic contribution to the development of CAAs and the presence of so-called therapy or IVIG resistance is also a topic of research (30–40).

Over the past decades research also aimed to find a singular ubiquitous pathogen, that could trigger the immune system in genetically pre-disposed children. The hypothesis of an infectious pathogen is supported by the epidemiologic features of KD, namely that young infants under the age of 6 months and adults very rarely develop KD (12, 41, 42). Young infants are protected by maternal antibodies, and adults already would have been exposed to this pathogen and protected by the adaptive immune system. Moreover, sudden peaks in incidence as observed in Japan, support such singular transmissible agents to evoke KD (43, 44). The current COVID-19 pandemic (caused by coronavirus SARS-Cov-2) also suggests that an infectious agent can, at least in some of the affected children, cause a syndrome similar or even identical with KD or KD-like shock syndrome (45–48). Despite extensive research, a common pathogen as the cause of KD has not been identified and a variety of pathogens have been found in KD patients in the past such as SARS-CoV-2, measles, human herpesvirus-6, parvovirus B19 and EBV (45, 49–55). Most likely the immune response causing KD can be triggered by multiple pathogens, as opposed to one single and specific pathogen.

Studies have suggested that the immune response in KD is mainly IgA-driven with a notable absence of IgM and IgG at the affected sites (41, 56, 57). Also high concentrations of IgA have been found at mucosal sites of the trachea of KD patients (56). These findings suggest that the upper respiratory tract could be the point of access for the pathogen to enter the body and consequently triggering KD, which may be associated with the local enlargement of often unilateral lymph nodes in the sternocleidomastoideus region. Studies relating wind patterns to the spread of KD further support the suggestion of an air-borne factor (58, 59). The higher prevalence of KD among Japanese, Taiwanese and children from South-East Asia as well as the familial cases and incidence among identical twins suggest a genetic risk, which suggest that an infectious trigger causes KD in genetically susceptible individuals. The genetic impact and its contribution to the risk to develop KD seems limited since the disease is particularly prevalent under the age of 5 (11). This suggests that development of immune reactivity plays an important role in the inherent susceptibility to the often undefined KD triggers.

An under-challenged immune system at an early age has previously been proven to pre-dispose children to specific diseases such as Acute Lymphoblastic Leukemia (60). This abnormal response to a common infection due to an under-challenged immune system has become known as the “Greaves” hypothesis (61, 62). According to this theory, a low exposure to common infections at a young age, could influence the development of the immune system and result in an altered development and maturation of the adaptive immune system. This would suggest that in the case of KD, not only nature (genetic factor) plays an important role in the pre-disposition of KD but also nurture (degree of exposure to common infections or environmental immune-reactive substances due to e.g., hygiene measures or living conditions) could play an important role to develop the disease at early age. A low exposure could facilitate a pathological response to a pathogen and triggering KD.

Cytomegalovirus (CMV) and Epstein-Barr virus (EBV) are both highly contagious viruses, which are commonly acquired during early childhood, and remain systemically suppressed in healthy individuals but ever-present, hence shaping or imprinting the immune reactivity of a given individual (63). CMV and EBV are a good reflection of viral exposure, as they spread through bodily fluids, as other commonly acquired viruses do (such as influenza, parvovirus etc.). Therefore, we used CMV and EBV as an indicator of the exposure state to systemic pathogens, reasoning that ever-present viruses would be beneficial to prepare the immune reactivity to subsequent infections. The aim of this study was to assess whether the incidence of CMV and EBV infection is lower in children with KD compared to a control pediatric population as a potential read-out for the under-challenged state of the immune system in those children affected by KD.

## Materials and Methods

### Patient Population

Based on the criteria of the American Heart Association (AHA) (64), patients with KD from the Dutch national referral center for KD were included. Retrospectively, we collected CMV and EBV antibody test results [IgG to EBNA (Epstein Barr nuclear antigen), IgG and IgM to EBV VCA (viral capsid antigen), and IgG and IgM to CMV] during follow up. After we excluded the patients with positive IgM to exclude any acute infections and the risk of cross-reactive serology results of EBV and CMV, we calculated seroprevalence. Clinical information was extracted from medical records. The blood samples taken within 9 months of IVIG treatment (or 10 months in case of a second IVIG treatment within a month of first treatment) were excluded. Samples taken later than 2 years after onset of disease and patients that were treated for a second episode of KD were excluded. Samples taken at the time of diagnosis, prior to IVIG infusion were included. We categorized the results according to age and we defined the following age groups: 0.5–2 years, 2–4 years, 4–6 years, 6–10 years, and 10–18 years.

### Control Group

We compared the age-dependent CMV- and EBV-seroprevalence in our KD patients to CMV- and EBV-seroprevalences in a control group. For this control group we used the requested EBV VCA IgG and IgM, as well as CMV IgG and IgM results (in the age group 6 months until 18 years) from the same laboratory as the one used by the national referral center from January 2000 until June 2019. As our hospital is a national referral center for KD, the follow-up of KD patients takes place exclusively within our KD team, therefore we were able to exclude KD patients from the control group by simply disqualifying the EBV VCA IgG and CMV IgG requested by our KD team members (physicians treating KD patients). We also excluded children from the control group with a positive IgM for CMV and/or EBV. Only the first requested CMV and EBV sample of a patient was included. If a laboratory result turned out to be inconclusive (borderline value or not enough blood drawn) this result was excluded, and a possible subsequent value was included instead. “Weak positive” results were regarded as positive. Finally we categorized the results according to age in the same categories as the KD group.

### Laboratory Analysis

Following the instructions of the manufacturers, IgG antibodies to EBV initially were determined using the anti-EBV VCA IgG, and EBNA IgG ELISAs from Biotest/BioRad Medical Diagnostics GmbH (Dreieich, Germany); later using the automated Liaison assays from DiaSorin (Saluggia, Italy); IgG to CMV initially was determined using the automated AxSym assays from Abbott Diagnostics (Chicago, USA); later using the automated Liaison assays from DiaSorin (Saluggia, Italy).

### Statistical Analysis

Our data consisted of a small sample size with a binomial distribution, therefore we performed a chi-squared test of independence using IBM Corp. Released 2016 SPSS Statistics for Windows, Version 24.0 to compare the seroprevalences in the Kawasaki group with their age- peers in the control group.

## Results

As shown in Table 1, the prevalence of CMV IgG was 6.7% in the KD group as compared to 36.2% in the control group in the 0.5–2 years age category; 21.4% compared to 36.7% in the 2–4 years old category; 26.3% compared to 35.1% in the 4–6 age group and 33.3% compared to 40.9% in the 6–10 years old category. *P-*values were 0.018, 0.098, 0.430, and 0.552, respectively. Similarly we found a lower prevalence of EBV VCA IgG in the KD group as compared to the control group i.e., 14.3% as compared to 28.0% in the age category 0.5–2 years; 20.7% compared to 43.9% in the 2–4 years old category; 17.6% compared to 51.9% in the 4–6 years old category and 25.0% compared to 59.7% in the 6–10 years old category. *P*-values were 0.257, 0,013, 0.005, and 0.005, respectively. When combined we also found a lower seroprevalence of CMV and EBV together, in the KD group compared to the control group, but these differences did not reach levels of statistical significance.

**Table 1:** Age-matched IgG seropositivity for CMV (and/or EBV anti-VCA) in KD cases vs. controls.

	**CMV+**					**EBV+**					**Both EBV+** **and CMV+** **at sampling**				
**Age at time of measurement (years)**	**KD**		**No KD**		***P*- value**	**KD**		**No KD**		***P*-value**	**KD**		**No KD**		***P*-value**
	***n***	**%**	***n***	**%**		***n***	**%**	***n***	**%**		***n***	**%**	***n***	**%**	
0.5–2	1/15	6.7	259/716	36.2	0.018	2/14	14.3	190/679	28.0	0.257	0/12	0.0	59/476	12.4	0.193^*^
2–4	6/28	21.4	322/877	36.7	0.098	6/29	20.7	410/934	43.9	0.013	4/27	14.8	142/691	20.5	0.468^*^
4–6	5/19	26.3	279/796	35.1	0.430	3/17	17.6	495/954	51.9	0.005	1/17	5.9	146/644	22.7	0.100
6–10	5/15	33.3	492/1202	40.9	0.552^*^	4/16	25.0	840/1406	59.7	0.005	1/15	6.7	275/986	28.4	0.063

* *Not enough power*.

**Figure 1:**
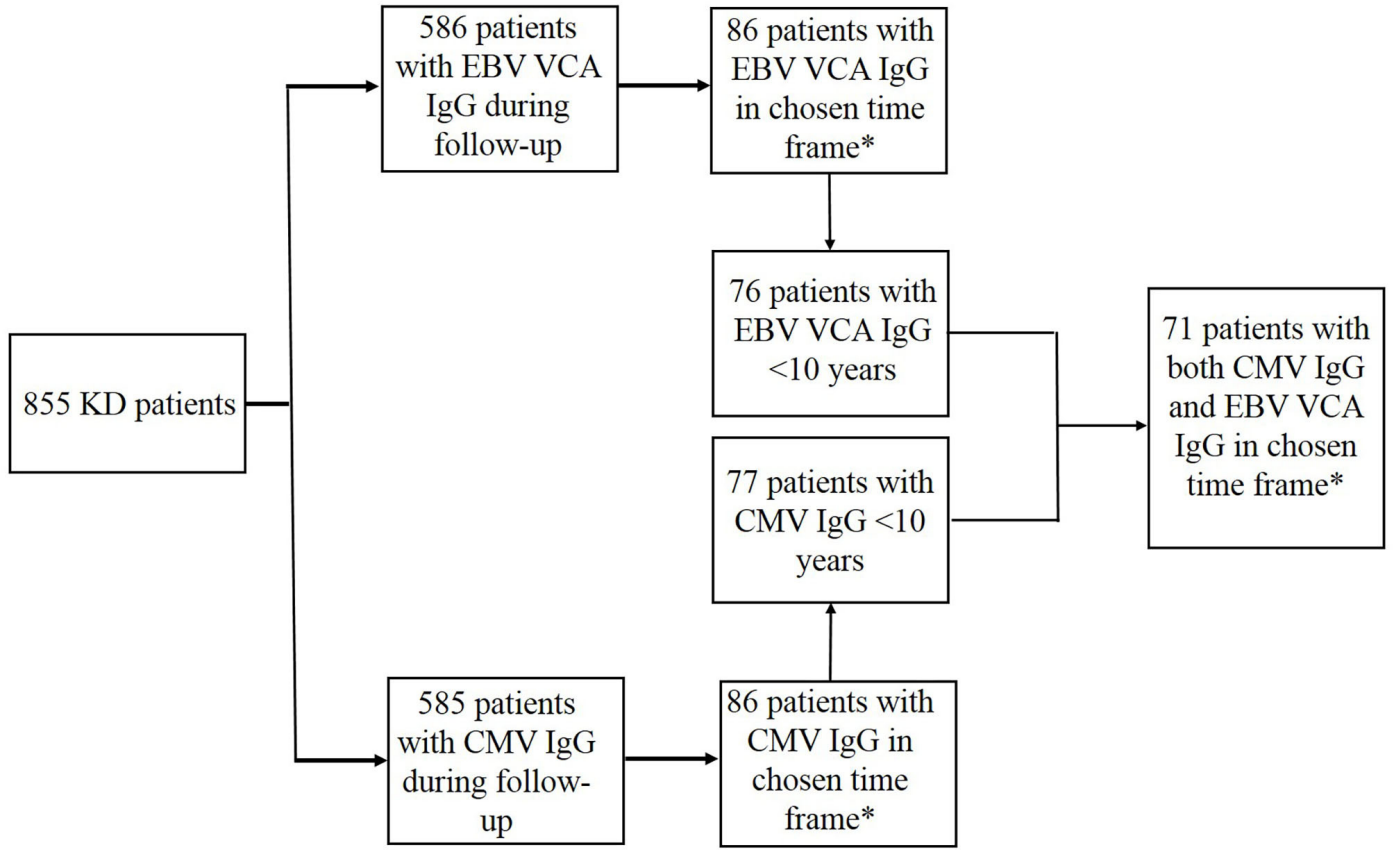
Patient selection. *Chosen time frame was 9 months after IVIG treatment but not later than 2 years after onset of disease.

From the 855 KD patients in our database 585 patients had CMV IgG and 586 patients had EBV VCA IgG determined during follow-up. From these patients, 86 patients had CMV IgG and another 86 patients had EBV VCA IgG determined within our chosen timeframe (Figure 1). The majority of the CMV IgG-tested KD patients (67.4%) was male and 8.1% had a partially reported Asian ethnicity. Similarly the majority of the EBV VCA IgG KD tested patients was male (68.6%) and 8.1% had a parental report of a mixed, partial Asian ethnicity (Table 2).

**Table 2:** Characteristics KD population.

	**CMV IgG tested**		**EBV VCA IgG tested**		**Both EBV VCA IgG and CMV IgG tested on the same date**	
	***n* (total = 86)**	**%**	***n* (total = 86)**	**%**	***n* (total = 77)**	**%**
**IVIG treatment**						
0.5–2 years	12 (15)	80.0	12 (14)	85.7	10 (12)	83.3
2–4 years	25 (28)	89.2	26 (29)	89.7	25 (27)	92.6
4–6 years	15 (19)	78.9	13 (17)	76.5	13 (17)	76.5
6–10 years	12 (15)	80.0	12 (16)	75.0	12 (15)	80.0
10–18 years	6 (9)	66.7	8 (10)	80.0	5 (6)	83.3
**Male gender**						
0.5–2 years	10 (15)	66.7	9 (14)	64.3	8 (12)	66.7
2–4 years	18 (28)	64.3	19 (29)	65.5	17 (27)	63.0
4–6 years	14 (19)	73.7	13 (17)	76.5	13 (17)	76.5
6–10 years	8 (15)	53.3	9 (16)	56.3	8 (15)	53.3
10–18 years	7 (9)	77.8	9 (10)	90.0	6 (6)	100
Complete KD^*^						
0.5–2 years	8 (15)	53.3	8 (14)	57.1	6 (12)	50.0
2–4 years	22 (28)	78.6	21 (29)	72.4	21 (27)	77.8
4–6 years	14 (19)	73.7	13 (17)	76.5	13 (17)	76.5
6–10 years	12 (15)	80.0	13 (16)	81.3	13 (15)	86.7
10–18 years	6 (9)	66.7	9 (10)	90.0	5 (6)	83.3
**No enlargement of coronary artery in acute phase (** ***Z*** **score** **<** 2.5)^**^						
0.5–2 years	10 (15)	66.7	9 (14)	64.3	6 (12)	50.0
2–4 years	19 (28)	67.9	20 (29)	68.9	18 (27)	66.7
4–6 years	14 (19)	73.7	12 (17)	70.5	12 (17)	70.6
6–10 years	11 (15)	73.3	12 (16)	75.0	11 (15)	73.3
10–18 years	5 (9)	55.6	6 (10)	60.0	4 (6)	66.7
**Enlargement of coronary artery in acute phase (** ***Z*** score 2.5–10)^**^						
0.5–2 years	3 (15)	20.0	3 (14)	21.4	3 (12)	25.0
2–4 years	2 (28)	7.1	2 (29)	10.5	2 (27)	7.4
4–6 years	3 (19)	15.8	3 (17)	17.6	3 (17)	17.6
6–10 years	1 (15)	6.7	1 (16)	6.25	1 (15)	6.7
10–18 years	1 (9)	11.1	1 (10)	10.0	1 (6)	16.7
**Giant aneurysm in acute phase (** ***Z*** **score** **≥** 10)^**^						
0.5–2 years	2 (15)	13.3	2 (14)	14.3	2 (12)	16.7
2–4 years	4 (28)	14.3	4 (29)	13.8	4 (27)	14.8
4–6 years	1 (19)	5.3	1 (17)	5.9	1 (17)	5.9
6–10 years	0 (15)	0	0 (16)	0	0 (15)	0
10–18 years	0 (9)	0	0 (10)	0	0 (6)	0
Dutch ethnicity^***^ **(both parents)**						
0.5–2 years	7 (15)	46.7	6 (14)	42.9	4 (12)	33.3
2–4 years	13 (28)	46.4	13 (29)	44.8	13 (27)	48.1
4–6 years	8 (19)	42.1	8 (17)	47.1	8 (17)	47.1
6–10 years	7 (15)	46.7	8 (16)	50.0	7 (15)	46.7
10–18 years	6 (9)	66.7	7 (10)	70.0	4 (6)	66.7
**(Partially) Asian ethnicity** ^***^, ^****^						
0.5–2 years	0 (15)	0	0 (14)	0	0 (12)	0
2–4 years	3 (28)	10.7	3 (29)	10.3	3 (27)	11.1
4–6 years	2 (19)	10.5	2 (17)	11.8	2 (17)	11.8
6–10 years	2 (15)	13.3	2 (16)	12.5	2 (15)	13.3
10–18 years	0 (9)	0	0 (10)	0	0 (6)	0
Other or unknown ethnicity^***^						
0.5–2 years	8 (15)	53.3	8 (14)	57.1	8 (12)	66.7
2–4 years	12 (28)	42.9	13 (29)	44.8	11 (27)	40.7
4–6 years	9 (19)	47.4	7 (17)	41.2	7 (17)	41.2
6–10 years	6 (15)	40.0	6 (16)	37.5	6 (15)	40.0
10–18 years	3 (9)	33.3	3 (10)	30.0	2 (6)	33.3

* *Complete KD is defined as: fever, combined with a minimum of four of the five other symptoms (rash, conjunctivitis, lymphadenopathy, changes of extremities and changes of the mouth). In 4 patients the symptoms were unknown*.

** *Z score was calculated using the McCrindle/Boston model*.

*** *Ethnicities were reported by parents/care-givers*.

**** *Asian countries that were included were: Vietnam, Japan, China, Indonesia and Thailand*.

After categorizing the age groups, there was insufficient data of KD children in the age category of >10 years, therefore we did not use this age group for our comparison. In the KD patients with CMV IgG measurements, 12 patients did not receive IVIG and 12 patients in whom IVIG was administered, the CMV IgG measurement took place at diagnosis of KD prior to IVIG (Table 3).

**Table 3:** Time between administration of IVIG and measurement of seroresponse (Δ Time).

	**Δ Time CMV IgG (median, range) in months**	**Δ Time EBV VCA IgG (median, range) in months**
**0.5–2 years**	14 (12–17), *n* = 8	14 (12–16), *n* = 9
Patients with measurement at diagnosis, prior to IVIG	*n* = 4	*n* = 3
Patients that did not receive IVIG	*n* = 3	*n* = 2
**2–4 years**	15 (10–22), *n* = 21	15 (10–22), *n* = 21
Patients with measurement at diagnosis, prior to IVIG	*n* = 4	*n* = 5
Patients that did not receive IVIG	*n* = 3	*n* = 3
**4–6 years**	14 (10–23), *n* = 13	14 (10–23), *n* = 12
Patients with measurement at diagnosis, prior to IVIG	*n* = 3	*n* = 2
Patients that did not receive IVIG	*n* = 3	*n* = 3
**6–10 years**	13 (10–22), *n* = 11	14.5 (10–22), *n* = 12
Patients with measurement at diagnosis, prior to IVIG	*n* = 1	*n* = 1
Patients that did not receive IVIG	*n* = 3	*n* = 3

Since we know that seroreactivity after administration of IVIG may last for more than 6–9 months (65) we assessed the median time that had passed between administration of IVIG and the measurement of the seroresponse of CMV IgG in the remaining KD patients which was 14 months (range 0–23) (Table 3). In the KD patients with EBV VCA IgG measurements, 11 patients did not receive IVIG and in 11 patients the EBV VCA IgG measurement took place at KD diagnosis prior to IVIG (Table 3). Also in the remainder of this group the median time that had passed between administration of IVIG and measurement of the seroresponse of EBV VCA IgG was 14 months (range 0–23).

**Figure 2:**
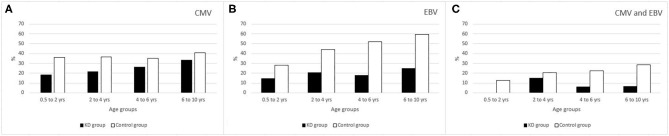
**(A)** % of CMV seropositivity. **(B)** % of EBV seropositivity. **(C)** % of CMV and EBV combined seropositivity.

For the control group, a total of 3114 EBV VCA IgG samples and 3591 CMV IgG samples were analyzed by the laboratory of by the national referral center. By comparison, the prevalence of CMV and EBV was lower in the KD population in all age groups tested against their age-matched peers of the control group (Figures 2A–C).

## Discussion

In the KD population, the prevalence of past CMV and EBV infection as indicated by seroprevalence, is lower than in an age-matched control group. This suggests that prior exposure to systemic infections by common viruses such as CMV and EBV might have a protective role in the susceptibility to KD at young age.

In the past, low exposure to infectious agents in early childhood has been proposed to increase the susceptibility to allergic diseases and autoimmune diseases due to the influence on the development of the immune system (66). Allergic reactions are known to be initiated by T helper lymphocytes while in KD a cellular immune dysfunction i.a. the imbalance of Th1 and Th2 or “split T cell anergy” has been suggested (67, 68). The theories about hygiene or the under-challenged immune system due to low exposure to environmental or infectious agents, have been suggested to contribute to a pathological response to a pathogen (69), which may be relevant to triggering KD in genetically susceptible children. Several studies have shown an increase of allergies in KD patients compared to the general population, which may support a link between hygiene and environmental conditions involved in the slow but progressive rise in KD incidence worldwide which can be neither caused by improved recognition and diagnostic skills of doctors nor by current laboratory tests for KD since these are not yet applicable (70–73).

An under-challenged immune system has previously been reported to have a causal connection to pre-dispose children to specific diseases such as Acute Lymphoblastic Leukemia (60, 61, 64). However, the majority of these studies used daycare attendance as an indicator for exposure to common infections, which is a very indirect measurement. Reasoning that at young age a viral infection could be such a trigger knowing that in the past several viral infections have been suggested as the cause of KD (49–54), we used CMV and EBV IgG seropositivity as an indication for exposure to common infections in this study. By excluding IgM positive results, we excluded the possibility of cross-reactive antibody results, which are a possible confounding factor if you look at an acute infection of these two viruses. Once infected, herpes viruses such as CMV and EBV stay lifelong present and do not cause any symptoms in healthy individuals, being suppressed by the immune system (63, 74, 75). Viral reactivation of herpes viruses may sometimes occur, especially during moments of reduced T-cell mediated immune suppression by concurrent disease such as malignancy, chronic disease, or the use of immunosuppressive medication, which then causes a raised alertness of the immune system against these viruses to keep them constrained. If immunity cannot suppress these reactivated viruses, the reactivated viruses may result in clinical disease. We have not observed any clinical reactivation as indicated by shingles or herpes zoster by herpes simplex virus (HSV) or varicella-zoster virus (VZV), respectively, or EBV or CMV DNA plasma concentrations during acute KD, indicating a globally intact immunity in these children.

When measuring seroprevalence of EBV and CMV, we observed a significant difference for EBV VCA IgG exposure in the KD group compared to the control group, except for the very young age category of 0.5–2 years old. The lack of significance could be explained by a generally low exposure to EBV in this age group and hence an underpowered comparison. In the same age group of 0.5–2 years old, there was a noticeable difference for CMV IgG seropositivity, which could be explained by higher contagiousness of CMV in early childhood and unnoticed transmission by breastfeeding, which may transmit CMV but far less commonly EBV (76–82). The differences in seroprevalance between EBV and CMV seem to decrease as age increases in our KD cohort. Of the positive EBV anti-VCA IgG measurements a few had anti-EBNA measurements (*n* = 12). Anti-EBNA antibodies are usually undetectable during the (sub)acute phase of the infection and slowly appear after onset of disease within the following weeks or months. Therefore, a positive anti-EBNA response indicates an infection in the past. From the anti-EBNA measurements most were negative (67%), with a median time of 14.5 months (range 11–18 months) between IVIG infusion and date of measurement. These infections could therefore be more recent, possibly after KD. But caution needs to be taken with the interpretation of anti-EBNA measurements due to the wide range in which even healthy controls can become positive. Of the cases in which both anti-EBNA and anti-VCA IgG were positive, the median time between IVIG and date of measurement was 14.5 months (range 11–18 months). We did not assess the anti-EBNA measurements in the control group due to the before mentioned limited value of these EBNA antibodies (83). The possibility of spurious IgG seropositive measurements because of prior IVIG infusion (65, 84) was excluded because of the time passed [14 months (range 0–23)] between the administration of IVIG and the measurement of the actual serology.

CMV is known to challenge the immune system, altering the immune response to other pathogens (85). Both EBV and CMV cause a redistribution in B-cell subsets (86–88). Herpes viruses like EBV and CMV causing systemic infection of the immune system and the lifelong survival within the host with transient virus reactivation from time to time—going most often completely unnoticed and without any clinical symptoms (74, 89–96) –, are expected to leave an imprint on the immune system because of their lifelong infection of that same system. This effect on the host's immune system could affect the response to a pathogen, also to the as yet unidentified and probably miscellaneous agents that may trigger KD. Although we cannot directly prove the hypothesis, our findings may suggest that insufficient priming of the immune system at an early age plays a role in the immune response in KD. Although we do not want to overemphasize a direct role in KD susceptibility for the two herpesviruses that we have tested, the rising incidence of KD in Japan, and the falling incidence of EBV at the same time (97) would support this theory on an under-challenged immune system. At the molecular level the immunological data currently available on epigenetic imprinting of T and B lymphocytes also indicate that previous infections impact the reactivity against subsequent triggers. These phenomena are definitely not considered as KD-specific or selective for certain age categories, but it supports the notion that prior exposure shapes the immunological outcome. Similar findings have been demonstrated at the epigenetic level of the innate immune system by exposure to mycobacterial components, which more recently has been a basis for BCG immunization to protect from Sars-CoV2 infection, as an example of “trained (innate) immunity” (98). The induction of a mature and experienced immune system to protect from a wide variety of triggers at young age may help to understand the otherwise unexplained incidence of KD in the under-5 years old while becoming almost zero at late adolescence (99, 100).

This is a study with intrinsic limitations, namely our control group might not be a representation of a completely healthy population due to the fact that the serological tests were requested by general practitioners and doctors from the hospital for KD-unrelated reasons. On the other hand, these anonymous controls were screened for infection similar to the children presenting with KD, for that reason being a cohort that might be more comparable with our KD children than a completely healthy population.

## Conclusion

We observed a significantly reduced CMV and EBV prevalence in KD patients compared to age-matched controls. The data suggest that an under-challenged immune system may contribute to the inflammatory vasculitis in children with an inherent polygenic and complex susceptibility to KD.

## Data Availability Statement

The original contributions generated in the study are included in the article/supplementary material, further inquiries can be directed to the corresponding author.

## Ethics Statement

Ethical review and approval was not required for the study on human participants in accordance with the local legislation and institutional requirements. Written informed consent to participate in this study was provided by the participants' legal guardian/next of kin.

## Author Contributions

DS conceptualized the study, collected data, interpreted data, and drafted the manuscript. AS collected data, performed the analysis, and contributed in the initial draft of the manuscript. TK and HZ coordinated and supervised data collection and reviewed the manuscript for important intellectual content and revised the manuscript. All authors approved the final manuscript as submitted and agree to be accountable for all aspects of the work.

## Conflict of Interest

The authors declare that the research was conducted in the absence of any commercial or financial relationships that could be construed as a potential conflict of interest.

## Abbreviations

CAAs: Coronary artery aneurysms
CMV: cytomegalovirus
EBV: Epstein-Barr virus
HSV: herpes simplex virus
IVIG: intravenous immunoglobulin
KD: Kawasaki disease
VZV: varicella-zoster virus.
