# Corrigendum: Lower CMV and EBV Exposure in Children With Kawasaki Disease Suggests an Under-Challenged Immune System

**DOI:** 10.3389/fped.2021.765546

**Published:** 2021-09-10

**Authors:** Diana van Stijn, Annemarie Slegers, Hans Zaaijer, Taco Kuijpers

**Affiliations:** ^1^Department of Pediatric Immunology, Rheumatology and Infectious Diseases, Emma Children's Hospital, Amsterdam University Medical Center, University of Amsterdam, Amsterdam, Netherlands; ^2^Laboratory of Clinical Virology, Department of Medical Microbiology, Center for Infection and Immunity Amsterdam University Medical Center, University of Amsterdam, Amsterdam, Netherlands

**Keywords:** Kawasaki disease, Epstein-Barr virus, immune system, viral exposure, cytomegalovirus

An author name was incorrectly spelled as Diana van Stijn-Bringas Dimitriades. The correct spelling is Diana van Stijn.

The authors apologize for this error and state that this does not change the scientific conclusions of the article in any way. The original article has been updated.

## Publisher's Note

All claims expressed in this article are solely those of the authors and do not necessarily represent those of their affiliated organizations, or those of the publisher, the editors and the reviewers. Any product that may be evaluated in this article, or claim that may be made by its manufacturer, is not guaranteed or endorsed by the publisher.

